# Diaqua­(5-methyl­pyrazine-2-carboxyl­ato-κ^2^ *N* ^1^,*O*)iron(II)

**DOI:** 10.1107/S1600536808042529

**Published:** 2008-12-20

**Authors:** Guang Fan, Jia-Juan Sun, Jun-Cai Zhang, Zhan-Ying Ma, Sheng-Li Gao

**Affiliations:** a Department of Chemistry, Xianyang Normal University, Xianyang 712000, Shaanxi, People’s Republic of China; b Department of Chemistry, Northwest University, Xi’an 710069, Shaanxi, People’s Republic of China

## Abstract

In the neutral title complex, [Fe(C_6_H_5_N_2_O_2_)_2_(H_2_O)_2_], the coordination geometry aound the Fe^II^ atom, which lies on an inversion centre, is distorted octa­hedral comprising two N atoms and two O atoms from two 5-methyl­pyrazine-2-carboxyl­ate ligands, and two water mol­ecules. The crystal structure is stabilized by a network of O—H⋯O hydrogen bonds, resulting in a two-dimensional supra­molecular structure.

## Related literature

For background to this study, see: Fan *et al.* (2007 ▶).
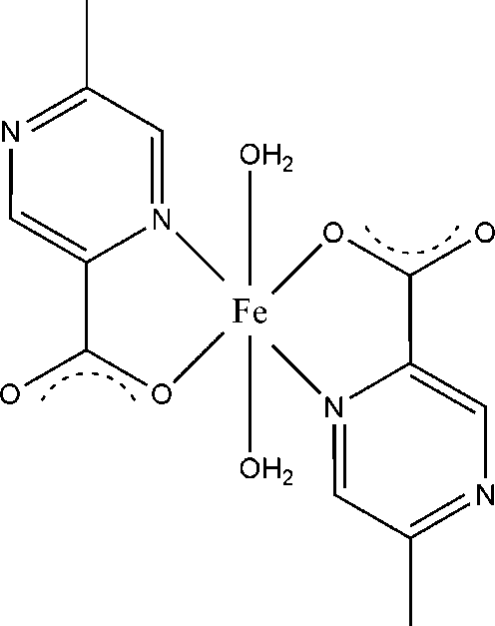


## Experimental

### 

#### Crystal data

[Fe(C_6_H_5_N_2_O_2_)_2_(H_2_O)_2_]
*M* *_r_* = 366.12
Triclinic, 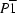

*a* = 5.068 (1) Å
*b* = 6.401 (1) Å
*c* = 12.381 (1) Å
α = 103.851 (2)°
β = 91.790(10)°
γ = 108.340 (2)°
*V* = 368.22 (10) Å^3^
*Z* = 1
Mo *K*α radiation
μ = 1.06 mm^−1^
*T* = 298 (2) K
0.18 × 0.09 × 0.05 mm

#### Data collection

Bruker SMART CCD area-detector diffractometer
Absorption correction: multi-scan (*SADABS*; Sheldrick, 1996 ▶) *T*_min_ = 0.832, *T*_max_ = 0.949
1916 measured reflections
1260 independent reflections
1061 reflections with *I* > 2σ(*I*)
*R*_int_ = 0.025

#### Refinement

*R*[*F* ^2^ > 2σ(*F* ^2^)] = 0.041
*wR*(*F* ^2^) = 0.095
*S* = 1.00
1260 reflections
107 parameters
H-atom parameters constrained
Δρ_max_ = 0.33 e Å^−3^
Δρ_min_ = −0.28 e Å^−3^

### 

Data collection: *SMART* (Bruker, 2002 ▶); cell refinement: *SAINT* (Bruker, 2002 ▶); data reduction: *SAINT*; program(s) used to solve structure: *SHELXS97* (Sheldrick, 2008 ▶); program(s) used to refine structure: *SHELXL97* (Sheldrick, 2008 ▶); molecular graphics: *SHELXTL* (Sheldrick, 2008 ▶); software used to prepare material for publication: *SHELXTL*.

## Supplementary Material

Crystal structure: contains datablocks I, global. DOI: 10.1107/S1600536808042529/ng2527sup1.cif

Structure factors: contains datablocks I. DOI: 10.1107/S1600536808042529/ng2527Isup2.hkl

Additional supplementary materials:  crystallographic information; 3D view; checkCIF report

## Figures and Tables

**Table 1:** Hydrogen-bond geometry (Å, °)

*D*—H⋯*A*	*D*—H	H⋯*A*	*D*⋯*A*	*D*—H⋯*A*
O3—H3*A*⋯O1^i^	0.85	1.93	2.720 (3)	155
O3—H3*B*⋯O2^ii^	0.85	1.86	2.673 (3)	159

Symmetry codes: (i) 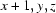; (ii) 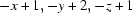.

**Fig. 1.**
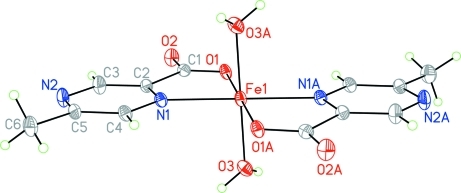
A view of the molecular structure of title complex with the atom-labling scheme. Displacement ellipsoids are drawn at the 30% probability level. Symmetry code for A: 1-X, 1-Y, 1-Z.

**Fig. 2.**
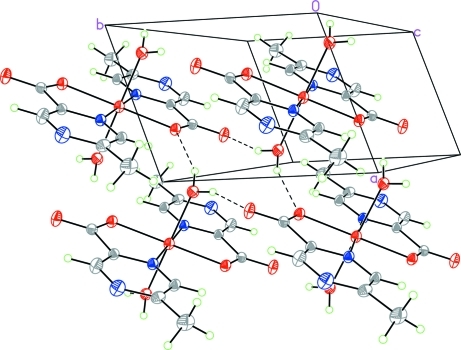
Two-dimensional supramolecular structure of the title complex.

## supplementary crystallographic information

### Comment

The background to this study is set out in the preceding paper (Fan *et al.*, 2007). Here we report the crystal structure of a mononuclear Fe^II^ (Fig. 1).

The asymmetric unit consists of a Fe^II^ atom, which lies on an inversion centre, one 2mpac ligand and two water molecules. A ring nitrogen atom and an oxygen atom of the carboxylate group from 2mpac ligand with Fe1—O1 = 2.103 (2) Å and Fe1—N1 = 2.167 (3) Å are involved in coordination to the Fe^II^ atom; these form a square. The coordination of the two water molecules with Fe1—O3 = 2.114 (2) Å occupied the axial sites results in the formation of a distorted octahedral geometry.

In the crystal structure, hydrogen bonding interactions are observed between the hydrogen atoms of the coordinated water molecules and the oxygen atoms of the carboxyl groups of a neighbouring unit, affording a two-dimensional supramolecular structure (Figure 2).

### Experimental

The title compound was obtained from the mixture of ferrous ammonium sulfate hexahydrate(0.10 g, 0.25 mmol), 5-methylpyrazine-2-carboxylic acid (0.70 g, 0.5 mmol) and distilled water (20 ml), which was placed at room temperature for two weeks and red single crystals were obtained finally.

### Refinement

All H atoms attached to C atoms from the organic ligands were generated in idealized positions and constrained to ride on their parent atoms, with d(C—H) = 0.93 Å, *U*_iso_=1.2*U*_eq_ (C) for aromatic and 0.96 Å, *U*_iso_ = 1.5*U*_eq_ (C) for CH_3_ atoms.

### Crystal data

**Table:**

[Fe(C_6_H_5_N_2_O_2_)_2_(H_2_O)_2_]	*Z* = 1
*M**_r_* = 366.12	*F*(000) = 188
Triclinic, *P*1	*D*_x_ = 1.651 Mg m^−^^3^
*a* = 5.068 (1) Å	Mo *K*α radiation, λ = 0.71073 Å
*b* = 6.401 (1) Å	Cell parameters from 715 reflections
*c* = 12.3810 (12) Å	θ = 3.4–26.8°
α = 103.851 (2)°	µ = 1.06 mm^−^^1^
β = 91.079 (1)°	*T* = 298 K
γ = 108.340 (2)°	Block, red
*V* = 368.22 (10) Å^3^	0.18 × 0.09 × 0.05 mm

### Data collection

**Table:**

Bruker SMART CCD area-detector diffractometer	1260 independent reflections
Radiation source: fine-focus sealed tube	1061 reflections with *I* > 2σ(*I*)
graphite	*R*_int_ = 0.025
φ and ω scans	θ_max_ = 25.0°, θ_min_ = 1.7°
Absorption correction: multi-scan (*SADABS*; Sheldrick, 1996)	*h* = −5→6
*T*_min_ = 0.832, *T*_max_ = 0.949	*k* = −7→7
1916 measured reflections	*l* = −14→12

### Refinement

**Table:**

Refinement on *F*^2^	Primary atom site location: structure-invariant direct methods
Least-squares matrix: full	Secondary atom site location: difference Fourier map
*R*[*F*^2^ > 2σ(*F*^2^)] = 0.041	Hydrogen site location: inferred from neighbouring sites
*wR*(*F*^2^) = 0.095	H-atom parameters constrained
*S* = 1.00	*w* = 1/[σ^2^(*F*_o_^2^) + (0.0456*P*)^2^] where *P* = (*F*_o_^2^ + 2*F*_c_^2^)/3
1260 reflections	(Δ/σ)_max_ < 0.001
107 parameters	Δρ_max_ = 0.33 e Å^−^^3^
0 restraints	Δρ_min_ = −0.28 e Å^−^^3^

### Special details

**Table:**

Geometry. All e.s.d.'s (except the e.s.d. in the dihedral angle between two l.s. planes) are estimated using the full covariance matrix. The cell e.s.d.'s are taken into account individually in the estimation of e.s.d.'s in distances, angles and torsion angles; correlations between e.s.d.'s in cell parameters are only used when they are defined by crystal symmetry. An approximate (isotropic) treatment of cell e.s.d.'s is used for estimating e.s.d.'s involving l.s. planes.
Refinement. Refinement of *F*^2^ against ALL reflections. The weighted *R*-factor *wR* and goodness of fit *S* are based on *F*^2^, conventional *R*-factors *R* are based on *F*, with *F* set to zero for negative *F*^2^. The threshold expression of *F*^2^ > σ(*F*^2^) is used only for calculating *R*-factors(gt) *etc*. and is not relevant to the choice of reflections for refinement. *R*-factors based on *F*^2^ are statistically about twice as large as those based on *F*, and *R*- factors based on ALL data will be even larger.

### Fractional atomic coordinates and isotropic or equivalent isotropic displacement parameters (Å^2^)

**Table:**

	*x*	*y*	*z*	*U*_iso_*/*U*_eq_	
Fe1	0.5000	0.5000	0.5000	0.0326 (3)	
N1	0.5923 (6)	0.4742 (4)	0.3281 (2)	0.0323 (7)	
N2	0.6791 (7)	0.4921 (6)	0.1096 (2)	0.0508 (8)	
O1	0.3162 (5)	0.7203 (4)	0.45178 (18)	0.0359 (6)	
O2	0.2550 (5)	0.8729 (4)	0.3133 (2)	0.0472 (7)	
O3	0.8721 (5)	0.7772 (4)	0.56033 (19)	0.0423 (6)	
H3A	1.0121	0.7975	0.5226	0.051*	
H3B	0.8701	0.9077	0.5961	0.051*	
C1	0.3463 (7)	0.7489 (5)	0.3543 (3)	0.0330 (8)	
C2	0.5049 (7)	0.6138 (5)	0.2826 (3)	0.0313 (8)	
C3	0.5494 (8)	0.6191 (7)	0.1744 (3)	0.0482 (10)	
H3	0.4856	0.7167	0.1445	0.058*	
C4	0.7211 (7)	0.3461 (6)	0.2642 (3)	0.0360 (8)	
H4	0.7855	0.2487	0.2940	0.043*	
C5	0.7624 (7)	0.3530 (6)	0.1548 (3)	0.0394 (8)	
C6	0.9003 (9)	0.2011 (7)	0.0829 (3)	0.0582 (11)	
H6A	0.7600	0.0722	0.0347	0.087*	
H6B	1.0059	0.1495	0.1297	0.087*	
H6C	1.0232	0.2847	0.0384	0.087*	

### Atomic displacement parameters (Å^2^)

**Table:**

	*U*^11^	*U*^22^	*U*^33^	*U*^12^	*U*^13^	*U*^23^
Fe1	0.0405 (5)	0.0352 (4)	0.0282 (4)	0.0197 (3)	0.0122 (3)	0.0092 (3)
N1	0.0366 (16)	0.0321 (16)	0.0329 (15)	0.0170 (13)	0.0109 (12)	0.0089 (13)
N2	0.064 (2)	0.068 (2)	0.0329 (17)	0.0347 (19)	0.0192 (15)	0.0171 (17)
O1	0.0419 (14)	0.0395 (14)	0.0356 (14)	0.0238 (11)	0.0170 (10)	0.0118 (11)
O2	0.0649 (18)	0.0445 (16)	0.0469 (15)	0.0355 (14)	0.0114 (12)	0.0151 (13)
O3	0.0427 (15)	0.0346 (14)	0.0527 (16)	0.0186 (12)	0.0178 (11)	0.0082 (12)
C1	0.0330 (19)	0.0275 (18)	0.037 (2)	0.0099 (15)	0.0054 (14)	0.0044 (15)
C2	0.0337 (19)	0.0296 (18)	0.0322 (19)	0.0137 (15)	0.0061 (14)	0.0066 (15)
C3	0.063 (3)	0.059 (3)	0.038 (2)	0.035 (2)	0.0160 (18)	0.020 (2)
C4	0.039 (2)	0.037 (2)	0.039 (2)	0.0206 (16)	0.0122 (16)	0.0104 (16)
C5	0.038 (2)	0.047 (2)	0.0333 (19)	0.0198 (17)	0.0102 (15)	0.0036 (17)
C6	0.065 (3)	0.063 (3)	0.047 (2)	0.030 (2)	0.019 (2)	−0.001 (2)

### Geometric parameters (Å, °)

**Table:**

Fe1—O1^i^	2.103 (2)	O3—H3A	0.8500
Fe1—O1	2.103 (2)	O3—H3B	0.8499
Fe1—O3^i^	2.114 (2)	C1—C2	1.510 (5)
Fe1—O3	2.114 (2)	C2—C3	1.369 (5)
Fe1—N1^i^	2.167 (3)	C3—H3	0.9300
Fe1—N1	2.167 (3)	C4—C5	1.384 (5)
N1—C4	1.331 (4)	C4—H4	0.9300
N1—C2	1.339 (4)	C5—C6	1.505 (5)
N2—C5	1.323 (4)	C6—H6A	0.9600
N2—C3	1.335 (5)	C6—H6B	0.9600
O1—C1	1.268 (4)	C6—H6C	0.9600
O2—C1	1.232 (4)		
			
O1^i^—Fe1—O1	180.00 (11)	H3A—O3—H3B	107.6
O1^i^—Fe1—O3^i^	89.75 (9)	O2—C1—O1	126.0 (3)
O1—Fe1—O3^i^	90.25 (9)	O2—C1—C2	117.9 (3)
O1^i^—Fe1—O3	90.25 (9)	O1—C1—C2	116.0 (3)
O1—Fe1—O3	89.75 (9)	N1—C2—C3	119.7 (3)
O3^i^—Fe1—O3	180.00 (10)	N1—C2—C1	116.5 (3)
O1^i^—Fe1—N1^i^	77.21 (9)	C3—C2—C1	123.7 (3)
O1—Fe1—N1^i^	102.79 (9)	N2—C3—C2	123.6 (3)
O3^i^—Fe1—N1^i^	92.02 (9)	N2—C3—H3	118.2
O3—Fe1—N1^i^	87.98 (9)	C2—C3—H3	118.2
O1^i^—Fe1—N1	102.79 (9)	N1—C4—C5	122.1 (3)
O1—Fe1—N1	77.21 (9)	N1—C4—H4	118.9
O3^i^—Fe1—N1	87.98 (9)	C5—C4—H4	118.9
O3—Fe1—N1	92.02 (9)	N2—C5—C4	121.0 (3)
N1^i^—Fe1—N1	180.000 (1)	N2—C5—C6	117.8 (3)
C4—N1—C2	117.2 (3)	C4—C5—C6	121.2 (3)
C4—N1—Fe1	130.3 (2)	C5—C6—H6A	109.5
C2—N1—Fe1	112.5 (2)	C5—C6—H6B	109.5
C5—N2—C3	116.3 (3)	H6A—C6—H6B	109.5
C1—O1—Fe1	117.6 (2)	C5—C6—H6C	109.5
Fe1—O3—H3A	121.1	H6A—C6—H6C	109.5
Fe1—O3—H3B	121.9	H6B—C6—H6C	109.5
			
O1^i^—Fe1—N1—C4	2.7 (3)	C4—N1—C2—C3	0.2 (5)
O1—Fe1—N1—C4	−177.3 (3)	Fe1—N1—C2—C3	179.5 (3)
O3^i^—Fe1—N1—C4	−86.6 (3)	C4—N1—C2—C1	177.0 (3)
O3—Fe1—N1—C4	93.4 (3)	Fe1—N1—C2—C1	−3.7 (3)
N1^i^—Fe1—N1—C4	131 (100)	O2—C1—C2—N1	−177.5 (3)
O1^i^—Fe1—N1—C2	−176.5 (2)	O1—C1—C2—N1	1.4 (4)
O1—Fe1—N1—C2	3.5 (2)	O2—C1—C2—C3	−0.9 (5)
O3^i^—Fe1—N1—C2	94.2 (2)	O1—C1—C2—C3	178.0 (3)
O3—Fe1—N1—C2	−85.8 (2)	C5—N2—C3—C2	1.1 (6)
N1^i^—Fe1—N1—C2	−48 (100)	N1—C2—C3—N2	−0.5 (6)
O1^i^—Fe1—O1—C1	29 (100)	C1—C2—C3—N2	−177.0 (3)
O3^i^—Fe1—O1—C1	−90.8 (2)	C2—N1—C4—C5	−0.6 (5)
O3—Fe1—O1—C1	89.2 (2)	Fe1—N1—C4—C5	−179.8 (2)
N1^i^—Fe1—O1—C1	177.1 (2)	C3—N2—C5—C4	−1.5 (5)
N1—Fe1—O1—C1	−2.9 (2)	C3—N2—C5—C6	177.9 (3)
Fe1—O1—C1—O2	−179.3 (3)	N1—C4—C5—N2	1.3 (5)
Fe1—O1—C1—C2	1.9 (4)	N1—C4—C5—C6	−178.0 (3)

Symmetry codes: (i) −*x*+1, −*y*+1, −*z*+1.

### Hydrogen-bond geometry (Å, °)

**Table:**

*D*—H···*A*	*D*—H	H···*A*	*D*···*A*	*D*—H···*A*
O3—H3A···O1^ii^	0.85	1.93	2.720 (3)	155
O3—H3B···O2^iii^	0.85	1.86	2.673 (3)	159

Symmetry codes: (ii) *x*+1, *y*, *z*; (iii) −*x*+1, −*y*+2, −*z*+1.
